# 9-[4-(Azido­meth­yl)phen­yl]-9*H*-carbazole-3-carbo­nitrile

**DOI:** 10.1107/S1600536814001391

**Published:** 2014-01-25

**Authors:** C. Ramathilagam, P. R. Umarani, N. Venkatesan, P. Rajakumar, B. Gunasekaran, V. Manivannan

**Affiliations:** a Department of Physics, AMET University, Kanathur, Chennai 603 112, India; b Principal, Kundavai Nachiyar Govt College for Women, Thanjavur 613 007, India; c Department of Organic Chemistry, University of Madras, Guindy Campus, Chennai 600 025, India; d Department of Physics & Nano Technology, SRM University, SRM Nagar, Kattankulathur, Kancheepuram Dist., Chennai 603 203, Tamil Nadu, India; e Department of Research and Development, PRIST University, Vallam, Thanjavur 613 403, Tamil Nadu, India

## Abstract

In the title compound C_20_H_13_N_5_, the dihedral angle between the carbazole ring system (r.m.s. deviation = 0.027 Å) and the pendant benzene ring is 55.08 (6)°. One of the azide N atoms is disordered over two positions in a 0.65 (2):0.35 (2) ratio. In the crystal, aromatic π–π stacking is observed [minimum centroid–centroid separation = 3.6499 (13) Å] as well as inversion-dimers connected by pairs of weak C—H⋯π inter­actions.

## Related literature   

For the biological activity of carbazole derivatives, see: Ramsewak *et al.* (1999 ▶); Tachibana *et al.* (2001 ▶); Itoigawa *et al.* (2000 ▶); Friend *et al.* (1999 ▶). For related structures, see: Velmurugan *et al.* (2010 ▶); Ramathilagam *et al.* (2011 ▶)
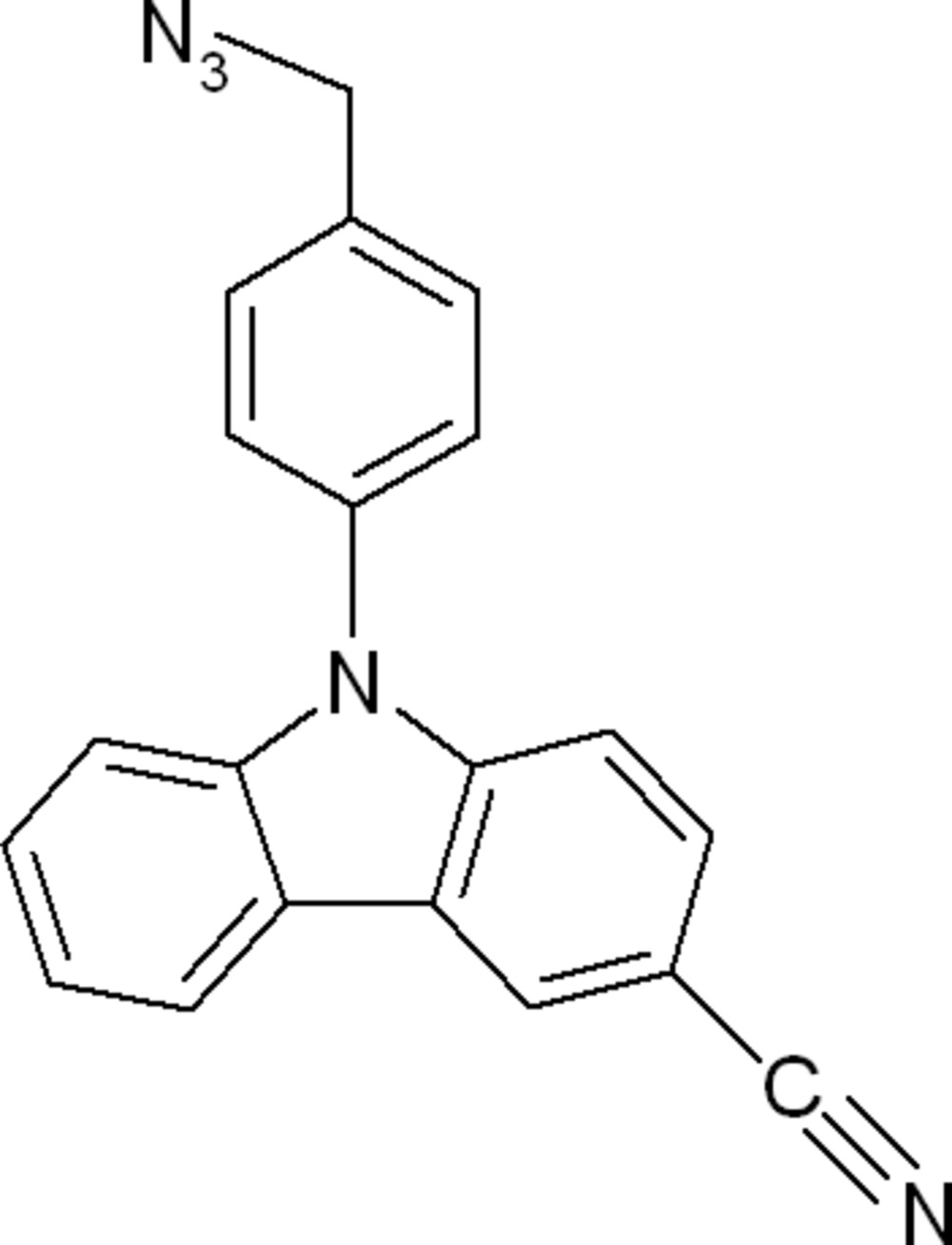


## Experimental   

### 

#### Crystal data  

C_20_H_13_N_5_
*M* *_r_* = 323.35
Monoclinic, 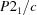

*a* = 9.8457 (2) Å
*b* = 8.4032 (2) Å
*c* = 19.9127 (5) Å
β = 90.970 (2)°
*V* = 1647.25 (7) Å^3^
*Z* = 4
Mo *K*α radiation
μ = 0.08 mm^−1^
*T* = 295 K
0.25 × 0.20 × 0.20 mm

#### Data collection  

Bruker kappa APEXII CCD diffractometer
Absorption correction: multi-scan (*SADABS*; Sheldrick, 1996 ▶) *T*_min_ = 0.958, *T*_max_ = 0.984
15375 measured reflections
4076 independent reflections
2676 reflections with *I* > 2σ(*I*)
*R*_int_ = 0.023

#### Refinement  

*R*[*F* ^2^ > 2σ(*F* ^2^)] = 0.065
*wR*(*F* ^2^) = 0.230
*S* = 1.07
4076 reflections
236 parameters
10 restraints
H-atom parameters constrained
Δρ_max_ = 0.67 e Å^−3^
Δρ_min_ = −0.32 e Å^−3^

### 

Data collection: *APEX2* (Bruker, 2008 ▶); cell refinement: *SAINT* (Bruker, 2008 ▶); data reduction: *SAINT*; program(s) used to solve structure: *SHELXS97* (Sheldrick, 2008 ▶); program(s) used to refine structure: *SHELXL97* (Sheldrick, 2008 ▶); molecular graphics: *PLATON* (Spek, 2009 ▶); software used to prepare material for publication: *SHELXL97*.

## Supplementary Material

Crystal structure: contains datablock(s) I. DOI: 10.1107/S1600536814001391/hb7185sup1.cif

Structure factors: contains datablock(s) I. DOI: 10.1107/S1600536814001391/hb7185Isup2.hkl

Supporting information file. DOI: 10.1107/S1600536814001391/hb7185Isup3.cml

CCDC reference:

Additional supporting information:  crystallographic information; 3D view; checkCIF report

## Figures and Tables

**Table 1:** Hydrogen-bond geometry (Å, °) *Cg*3 is the centroid of the C7–C12 ring.

*D*—H⋯*A*	*D*—H	H⋯*A*	*D*⋯*A*	*D*—H⋯*A*
C14—H14⋯*Cg*3^i^	0.93	2.91	3.673 (2)	140

Symmetry code: (i) 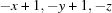.

**Fig. 1.**
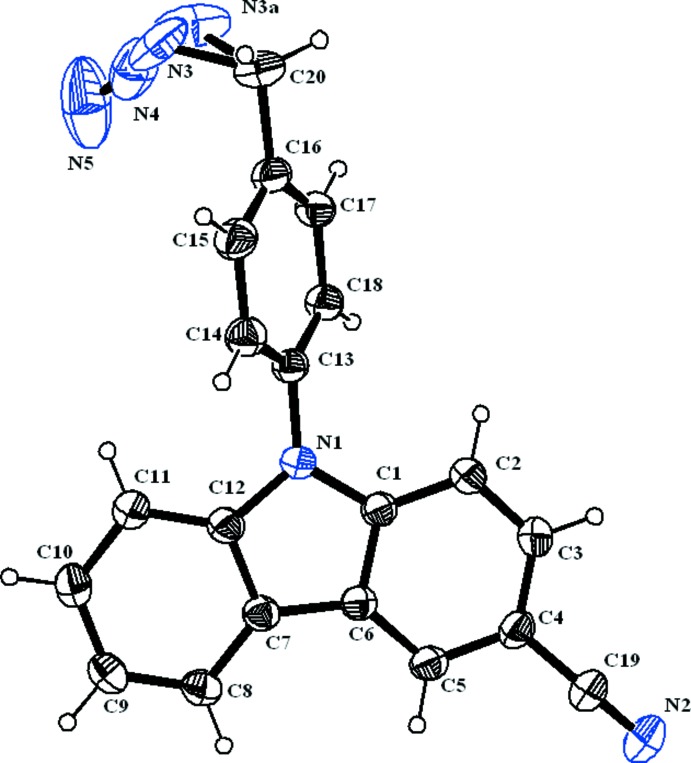
The molecular structure of (I), with 30% probability displacement ellipsoids for non-H atoms.

## supplementary crystallographic information

### 1. Comment

Carbazole derivatives possess antioxidative (Tachibana *et al.*, 2001), antitumor (Itoigawa *et al.*, 2000), anti-inflammatory and antimutagenic (Ramsewak *et al.*, 1999)activities. These derivatives also exhibit electroactivity and luminescence properties and are considered to be potential candidates for electronics such as colour display, organic semiconductor lasers and solar cells (Friend *et al.*, 1999).

The geometric parameters of the title molecule (Fig. 1) agree well with reported similar structure(Velmurugan *et al.*, 2010; Ramathilagam *et al.*, 2011). Phenyl ring makes the dihedral angle of 55.08 (6) ° with the carbazole ring system. In azidomethyl group one of the nitrogen atom is disordered over two position with site occupancies of 0.35 (2) and 0.65 (2). The sum of bond angles around the atom N1 [359.81 (17) °] indicates *sp^2^* hybridized. The crystal packing features weak C—H···π [C14—H14···*Cg*3(1 - *x*, 1 - *y*, -*z*) distance of 3.672 (7) Å, (*Cg*3 and is the centroid of the ring defined by the atoms C7—C12)] interactions.

### 2. Experimental

9-(4-(bromomethyl)phenyl)-9*H*-carbazole-3-carbonitrile (1.0 mmol, 1.0 equiv) was dissolved in acetone/water (4:1, 8 ml). NaN3 (1.5 mmol, 1.5 equiv.) was added and the mixture was heated at 60 ° C for 5 h. The reaction mixture was cooled to room temperature, acetone was evaporated and the reaction mixture was diluted with H2O (100 ml) and extracted with CHCl3 (2 *x* 100 ml). The organic layer was washed with saturated NaCl (50 ml), dried over Na2SO4 and evaporated gave 9-(4-(azidomethyl)phenyl) -9*H*-carbazole-3-carbonitrile as pale yellow blocks with a yield of 84% and melting point 148 ° C.

### 3. Refinement

In azidomethyl group one of the nitrogen atom is disordered over two position. The site occupancy factors of disordered nitrogen atoms were refined as N3 = 0.35 (2) and N3A = 0.65 (2) during anisotropic refinement. The N3A—N4 bond distance was restrained to be 1.1500 (1) Å. The atom N3A exhibited elongation of the thermal elipsoid and have been restrained (ISOR) to be more isotropic. The components of the anisotropic displacement parameters in direction of the bond N3—N4 and N4—N5, were restrained to be equal within an effective standard deviation of 0.001 using the DELU command in *SHELXL* (Sheldrick, 2008). H atoms were positioned geometrically and refined using riding model with C—H = 0.93 Å and *U*_iso_(H) = 1.2Ueq(C) for aromatic C—H, C—H = 0.97 Å and *U*_iso_(H) = 1.2Ueq(C) for CH2.

### Crystal data

**Table:**

C_20_H_13_N_5_	*F*(000) = 672
*M**_r_* = 323.35	*D*_x_ = 1.304 Mg m^−^^3^
Monoclinic, *P*2_1_/*c*	Mo *K*α radiation, λ = 0.71073 Å
Hall symbol: -P 2ybc	Cell parameters from 4076 reflections
*a* = 9.8457 (2) Å	θ = 2.0–28.3°
*b* = 8.4032 (2) Å	µ = 0.08 mm^−^^1^
*c* = 19.9127 (5) Å	*T* = 295 K
β = 90.970 (2)°	Block, pale yellow
*V* = 1647.25 (7) Å^3^	0.25 × 0.20 × 0.20 mm
*Z* = 4	

### Data collection

**Table:**

Bruker kappa APEXII CCD diffractometer	4076 independent reflections
Radiation source: fine-focus sealed tube	2676 reflections with *I* > 2σ(*I*)
Graphite monochromator	*R*_int_ = 0.023
Detector resolution: 0 pixels mm^-1^	θ_max_ = 28.3°, θ_min_ = 2.1°
ω and φ scans	*h* = −12→13
Absorption correction: multi-scan (*SADABS*; Sheldrick, 1996)	*k* = −10→11
*T*_min_ = 0.958, *T*_max_ = 0.984	*l* = −26→26
15375 measured reflections	

### Refinement

**Table:**

Refinement on *F*^2^	Primary atom site location: structure-invariant direct methods
Least-squares matrix: full	Secondary atom site location: difference Fourier map
*R*[*F*^2^ > 2σ(*F*^2^)] = 0.065	Hydrogen site location: inferred from neighbouring sites
*wR*(*F*^2^) = 0.230	H-atom parameters constrained
*S* = 1.07	*w* = 1/[σ^2^(*F*_o_^2^) + (0.1219*P*)^2^ + 0.4961*P*] where *P* = (*F*_o_^2^ + 2*F*_c_^2^)/3
4076 reflections	(Δ/σ)_max_ < 0.001
236 parameters	Δρ_max_ = 0.67 e Å^−^^3^
10 restraints	Δρ_min_ = −0.32 e Å^−^^3^

### Fractional atomic coordinates and isotropic or equivalent isotropic displacement parameters (Å^2^)

**Table:**

	*x*	*y*	*z*	*U*_iso_*/*U*_eq_	Occ. (<1)
C1	0.1427 (2)	0.6443 (3)	−0.02437 (10)	0.0462 (5)	
C2	0.0810 (2)	0.7908 (3)	−0.03579 (11)	0.0532 (5)	
H2	0.0891	0.8728	−0.0046	0.064*	
C3	0.0079 (3)	0.8108 (3)	−0.09424 (12)	0.0584 (6)	
H3	−0.0355	0.9073	−0.1025	0.070*	
C4	−0.0026 (2)	0.6891 (3)	−0.14171 (11)	0.0554 (6)	
C5	0.0600 (2)	0.5431 (3)	−0.13083 (10)	0.0502 (5)	
H5	0.0533	0.4627	−0.1628	0.060*	
C6	0.1326 (2)	0.5191 (2)	−0.07139 (10)	0.0453 (5)	
C7	0.2076 (2)	0.3867 (3)	−0.04382 (11)	0.0473 (5)	
C8	0.2302 (2)	0.2308 (3)	−0.06541 (12)	0.0541 (5)	
H8	0.1961	0.1962	−0.1067	0.065*	
C9	0.3039 (2)	0.1295 (3)	−0.02440 (13)	0.0602 (6)	
H9	0.3202	0.0258	−0.0384	0.072*	
C10	0.3538 (3)	0.1802 (3)	0.03750 (13)	0.0638 (6)	
H10	0.4019	0.1089	0.0646	0.077*	
C11	0.3343 (3)	0.3342 (3)	0.06016 (12)	0.0591 (6)	
H11	0.3687	0.3677	0.1016	0.071*	
C12	0.2609 (2)	0.4363 (3)	0.01842 (11)	0.0491 (5)	
C13	0.2663 (2)	0.6895 (2)	0.08503 (10)	0.0470 (5)	
C14	0.4037 (2)	0.7025 (3)	0.09936 (12)	0.0559 (6)	
H14	0.4666	0.6511	0.0726	0.067*	
C15	0.4469 (3)	0.7928 (3)	0.15393 (13)	0.0607 (6)	
H15	0.5393	0.8000	0.1640	0.073*	
C16	0.3556 (3)	0.8719 (3)	0.19349 (11)	0.0600 (6)	
C17	0.2184 (3)	0.8588 (3)	0.17812 (11)	0.0585 (6)	
H17	0.1556	0.9119	0.2044	0.070*	
C18	0.1733 (2)	0.7679 (3)	0.12418 (11)	0.0535 (5)	
H18	0.0809	0.7596	0.1144	0.064*	
C19	−0.0782 (3)	0.7148 (3)	−0.20322 (13)	0.0720 (7)	
C20	0.4028 (4)	0.9678 (4)	0.25333 (15)	0.0909 (10)	
H20A	0.4985	0.9911	0.2479	0.109*	
H20B	0.3548	1.0686	0.2523	0.109*	
N1	0.22210 (19)	0.5936 (2)	0.02970 (9)	0.0501 (5)	
N2	−0.1372 (4)	0.7353 (4)	−0.25201 (14)	0.1079 (10)	
N3	0.4560 (12)	0.8595 (18)	0.3064 (5)	0.106 (4)	0.35 (2)
N3A	0.3867 (14)	0.9011 (9)	0.3169 (3)	0.134 (4)	0.65 (2)
N4	0.3659 (4)	0.7676 (7)	0.32487 (16)	0.1170 (13)	
N5	0.3230 (6)	0.6426 (7)	0.3378 (3)	0.186 (3)	

### Atomic displacement parameters (Å^2^)

**Table:**

	*U*^11^	*U*^22^	*U*^33^	*U*^12^	*U*^13^	*U*^23^
C1	0.0527 (11)	0.0444 (11)	0.0414 (10)	−0.0050 (9)	−0.0002 (8)	0.0002 (8)
C2	0.0671 (13)	0.0423 (12)	0.0502 (12)	−0.0001 (10)	−0.0010 (10)	−0.0016 (9)
C3	0.0714 (15)	0.0498 (13)	0.0538 (12)	0.0052 (11)	−0.0009 (11)	0.0063 (10)
C4	0.0656 (14)	0.0580 (14)	0.0425 (11)	−0.0009 (10)	−0.0018 (10)	0.0077 (9)
C5	0.0591 (12)	0.0503 (12)	0.0413 (10)	−0.0080 (9)	0.0015 (9)	−0.0012 (9)
C6	0.0499 (11)	0.0430 (11)	0.0431 (10)	−0.0062 (8)	0.0037 (8)	−0.0002 (8)
C7	0.0513 (11)	0.0444 (11)	0.0462 (11)	−0.0047 (8)	0.0020 (8)	−0.0014 (8)
C8	0.0584 (12)	0.0478 (12)	0.0561 (13)	−0.0058 (10)	0.0016 (10)	−0.0078 (10)
C9	0.0661 (14)	0.0428 (12)	0.0717 (15)	0.0011 (10)	0.0036 (12)	−0.0033 (10)
C10	0.0712 (15)	0.0511 (14)	0.0688 (15)	0.0082 (11)	−0.0046 (12)	0.0065 (11)
C11	0.0697 (14)	0.0531 (14)	0.0540 (13)	0.0034 (11)	−0.0085 (11)	0.0006 (10)
C12	0.0547 (11)	0.0431 (11)	0.0496 (11)	−0.0034 (9)	0.0000 (9)	−0.0013 (9)
C13	0.0587 (12)	0.0416 (11)	0.0405 (10)	−0.0067 (9)	−0.0041 (8)	−0.0005 (8)
C14	0.0579 (13)	0.0554 (13)	0.0543 (12)	−0.0036 (10)	0.0004 (10)	−0.0017 (10)
C15	0.0632 (14)	0.0579 (14)	0.0606 (13)	−0.0114 (11)	−0.0123 (11)	0.0004 (11)
C16	0.0848 (17)	0.0479 (13)	0.0468 (12)	−0.0102 (11)	−0.0128 (11)	−0.0002 (9)
C17	0.0772 (16)	0.0525 (13)	0.0458 (12)	0.0007 (11)	0.0028 (11)	−0.0061 (10)
C18	0.0587 (12)	0.0504 (13)	0.0512 (12)	−0.0028 (10)	−0.0015 (10)	−0.0019 (10)
C19	0.096 (2)	0.0680 (17)	0.0518 (14)	0.0063 (14)	−0.0078 (13)	0.0073 (12)
C20	0.123 (3)	0.083 (2)	0.0659 (18)	−0.0031 (18)	−0.0312 (17)	−0.0208 (15)
N1	0.0608 (11)	0.0419 (10)	0.0472 (10)	0.0001 (8)	−0.0076 (8)	−0.0047 (7)
N2	0.157 (3)	0.102 (2)	0.0629 (15)	0.021 (2)	−0.0350 (17)	0.0106 (15)
N3	0.074 (5)	0.200 (7)	0.044 (5)	0.028 (5)	−0.013 (3)	−0.012 (5)
N3A	0.157 (8)	0.172 (6)	0.073 (3)	−0.066 (5)	0.018 (4)	−0.039 (4)
N4	0.112 (3)	0.176 (4)	0.0636 (17)	0.046 (3)	0.0171 (17)	0.027 (2)
N5	0.194 (5)	0.181 (4)	0.187 (5)	0.079 (3)	0.087 (4)	0.105 (4)

### Geometric parameters (Å, º)

**Table:**

C1—N1	1.387 (3)	C12—N1	1.395 (3)
C1—C2	1.389 (3)	C13—C18	1.380 (3)
C1—C6	1.411 (3)	C13—C14	1.382 (3)
C2—C3	1.368 (3)	C13—N1	1.427 (3)
C2—H2	0.9300	C14—C15	1.386 (3)
C3—C4	1.396 (3)	C14—H14	0.9300
C3—H3	0.9300	C15—C16	1.377 (4)
C4—C5	1.389 (3)	C15—H15	0.9300
C4—C19	1.439 (3)	C16—C17	1.385 (4)
C5—C6	1.387 (3)	C16—C20	1.505 (3)
C5—H5	0.9300	C17—C18	1.385 (3)
C6—C7	1.439 (3)	C17—H17	0.9300
C7—C8	1.397 (3)	C18—H18	0.9300
C7—C12	1.401 (3)	C19—N2	1.137 (4)
C8—C9	1.378 (3)	C20—N3A	1.397 (8)
C8—H8	0.9300	C20—N3	1.483 (11)
C9—C10	1.387 (4)	C20—H20A	0.9700
C9—H9	0.9300	C20—H20B	0.9700
C10—C11	1.385 (4)	N3—N4	1.237 (14)
C10—H10	0.9300	N3A—N4	1.1513 (10)
C11—C12	1.389 (3)	N4—N5	1.163 (7)
C11—H11	0.9300		
			
N1—C1—C2	129.68 (19)	C18—C13—C14	120.1 (2)
N1—C1—C6	108.53 (18)	C18—C13—N1	120.60 (19)
C2—C1—C6	121.78 (19)	C14—C13—N1	119.3 (2)
C3—C2—C1	118.0 (2)	C13—C14—C15	119.5 (2)
C3—C2—H2	121.0	C13—C14—H14	120.3
C1—C2—H2	121.0	C15—C14—H14	120.3
C2—C3—C4	121.2 (2)	C16—C15—C14	121.2 (2)
C2—C3—H3	119.4	C16—C15—H15	119.4
C4—C3—H3	119.4	C14—C15—H15	119.4
C5—C4—C3	120.9 (2)	C15—C16—C17	118.6 (2)
C5—C4—C19	119.2 (2)	C15—C16—C20	121.1 (3)
C3—C4—C19	119.9 (2)	C17—C16—C20	120.3 (3)
C6—C5—C4	118.9 (2)	C16—C17—C18	120.9 (2)
C6—C5—H5	120.6	C16—C17—H17	119.5
C4—C5—H5	120.6	C18—C17—H17	119.5
C5—C6—C1	119.2 (2)	C13—C18—C17	119.6 (2)
C5—C6—C7	133.7 (2)	C13—C18—H18	120.2
C1—C6—C7	107.10 (18)	C17—C18—H18	120.2
C8—C7—C12	119.5 (2)	N2—C19—C4	179.6 (4)
C8—C7—C6	133.7 (2)	N3A—C20—C16	117.7 (3)
C12—C7—C6	106.80 (19)	N3—C20—C16	109.6 (6)
C9—C8—C7	118.8 (2)	N3A—C20—H20A	107.9
C9—C8—H8	120.6	N3—C20—H20A	82.6
C7—C8—H8	120.6	C16—C20—H20A	107.9
C8—C9—C10	120.8 (2)	N3A—C20—H20B	107.9
C8—C9—H9	119.6	N3—C20—H20B	135.8
C10—C9—H9	119.6	C16—C20—H20B	107.9
C11—C10—C9	121.8 (2)	H20A—C20—H20B	107.2
C11—C10—H10	119.1	C1—N1—C12	108.59 (17)
C9—C10—H10	119.1	C1—N1—C13	125.89 (18)
C10—C11—C12	117.1 (2)	C12—N1—C13	125.34 (18)
C10—C11—H11	121.4	N4—N3—C20	110.4 (7)
C12—C11—H11	121.4	N4—N3A—C20	122.6 (6)
C11—C12—N1	129.1 (2)	N3A—N4—N5	167.6 (8)
C11—C12—C7	121.9 (2)	N5—N4—N3	153.5 (8)
N1—C12—C7	108.97 (19)		
			
N1—C1—C2—C3	179.5 (2)	C14—C15—C16—C17	0.5 (4)
C6—C1—C2—C3	0.7 (3)	C14—C15—C16—C20	179.1 (2)
C1—C2—C3—C4	−1.1 (4)	C15—C16—C17—C18	0.1 (4)
C2—C3—C4—C5	0.5 (4)	C20—C16—C17—C18	−178.4 (2)
C2—C3—C4—C19	−178.9 (2)	C14—C13—C18—C17	−0.4 (3)
C3—C4—C5—C6	0.6 (3)	N1—C13—C18—C17	179.3 (2)
C19—C4—C5—C6	−180.0 (2)	C16—C17—C18—C13	−0.2 (3)
C4—C5—C6—C1	−1.0 (3)	C15—C16—C20—N3A	−103.0 (8)
C4—C5—C6—C7	179.6 (2)	C17—C16—C20—N3A	75.6 (8)
N1—C1—C6—C5	−178.63 (18)	C15—C16—C20—N3	−69.1 (6)
C2—C1—C6—C5	0.4 (3)	C17—C16—C20—N3	109.5 (6)
N1—C1—C6—C7	0.9 (2)	C2—C1—N1—C12	180.0 (2)
C2—C1—C6—C7	179.95 (19)	C6—C1—N1—C12	−1.1 (2)
C5—C6—C7—C8	−3.3 (4)	C2—C1—N1—C13	−4.8 (4)
C1—C6—C7—C8	177.3 (2)	C6—C1—N1—C13	174.15 (19)
C5—C6—C7—C12	179.1 (2)	C11—C12—N1—C1	−176.2 (2)
C1—C6—C7—C12	−0.4 (2)	C7—C12—N1—C1	0.9 (2)
C12—C7—C8—C9	0.5 (3)	C11—C12—N1—C13	8.5 (4)
C6—C7—C8—C9	−176.9 (2)	C7—C12—N1—C13	−174.42 (19)
C7—C8—C9—C10	0.6 (4)	C18—C13—N1—C1	57.2 (3)
C8—C9—C10—C11	−1.1 (4)	C14—C13—N1—C1	−123.1 (2)
C9—C10—C11—C12	0.6 (4)	C18—C13—N1—C12	−128.3 (2)
C10—C11—C12—N1	177.2 (2)	C14—C13—N1—C12	51.3 (3)
C10—C11—C12—C7	0.5 (4)	N3A—C20—N3—N4	50.2 (7)
C8—C7—C12—C11	−1.0 (3)	C16—C20—N3—N4	−61.0 (11)
C6—C7—C12—C11	177.0 (2)	N3—C20—N3A—N4	−66.7 (13)
C8—C7—C12—N1	−178.34 (19)	C16—C20—N3A—N4	16.2 (17)
C6—C7—C12—N1	−0.3 (2)	C20—N3A—N4—N5	−118 (3)
C18—C13—C14—C15	1.0 (3)	C20—N3A—N4—N3	68.5 (11)
N1—C13—C14—C15	−178.6 (2)	C20—N3—N4—N3A	−51.9 (9)
C13—C14—C15—C16	−1.1 (4)	C20—N3—N4—N5	131.1 (12)

### Hydrogen-bond geometry (Å, º)

Cg3 is the centroid of the C7–C12 ring.

**Table:**

*D*—H···*A*	*D*—H	H···*A*	*D*···*A*	*D*—H···*A*
C14—H14···*Cg*3^i^	0.93	2.91	3.673 (2)	140

Symmetry code: (i) −*x*+1, −*y*+1, −*z*.
